# Clinical and Sociodemographic Predictors of Mortality in End-Stage Renal Disease Inpatients in Rural Areas of the USA: Evidence From the Nationwide Inpatient Sample

**DOI:** 10.7759/cureus.25624

**Published:** 2022-06-03

**Authors:** Emmanuel Adeyemi, Andrew Okpe, Chinedum Enete, Kim Dixon

**Affiliations:** 1 Internal Medicine, Saint Peter’s University Hospital, New Brunswick, USA

**Keywords:** end-stage renal disease (esrd), racial disparity, medicare funding, charlson comorbidity index, ckd (chronic kidney disease)

## Abstract

Background: End-stage renal disease (ESRD) has been associated with an increase in all-cause mortality among patients. The accumulation of comorbidities appears to be a contributing factor. This study set out to identify the effect of comorbidity severity and other predictors of mortality among ESRD inpatients in rural America.

Methods: This is a cross-sectional study that used the 2016-2018 Nationwide Inpatient Sample (NIS) from the Healthcare Cost and Utilization Project (HCUP). The study included patients aged 18 years or older with ESRD hospitalized in rural hospitals in America. Independent variables used in the survey include age, gender, race, type of admission (elective versus nonelective), type of hospital control, expected primary payer, and severity of comorbidities. The dependent variable was death during hospitalization. All analyses were weighted. Univariate (frequencies), bivariate (Chi-square), and logistic regression analyses were done using the SAS Studio (SAS Institute Inc., Cary, NC, USA).

Results: There were 144,575 weighted ESRD hospitalizations, and 5% of the hospitalized patients died. In the bivariate analysis, significant variables include age group, race, type of hospital admission, expected primary payer, type of hospital control, and severity of comorbidities, and all had a significant P-value of <0.0001. On multivariable logistic regression analysis, middle-aged and elderly patients had 40% (adjusted odds ratio (AOR): 1.40, 95% confidence interval (CI): 1.20-1.62) and 201% (AOR: 3.01, 95% CI: 2.61-3.48) more odds of mortality while hospitalized, respectively, compared to the young. Compared to whites, blacks had 19% (AOR: 0.81, 95% CI: 0.77-0.86) reduced odds of mortality, Hispanics had 47% (AOR: 0.53, 95% CI: 0.46-0.61) reduced odds of mortality, Native Americans had 27% (AOR: 0.73, 95% CI: 0.63-0.84) reduced odds of mortality, and Asian or Pacific Islanders had 30% (AOR: 0.70, 95% CI: 0.54-0.90) reduced odds of mortality. ESRD patients on nonelective hospitalizations had 16% (AOR: 0.84, 95% CI: 0.79-0.90) reduced odds of mortality while hospitalized versus those on elective hospitalization. ESRD patients with severe comorbidities had 40% (AOR: 1.40, 95% CI: 1.26-1.54) more odds of mortality compared to those with mild comorbidities, and those with moderate comorbidities had 22% (AOR: 1.22, 95% CI: 1.10-1.36) compared to those with mild comorbidities. Compared to patients on Medicare, ESRD hospitalizations on Medicaid had 19% (AOR: 1.19, 95% CI: 1.06-1.32) higher odds of mortality, hospitalizations on private insurance had 26% (AOR: 1.26, 96% CI: 1.15-1.37) higher odds of mortality, self-pay patients had 99% (AOR: 1.99, 95% CI: 1.61-2.45) higher odds of mortality, and no charge patients had over 1400% (AOR: 15.61, 95% CI: 7.09-34.35) higher odds of mortality. The area under the curve (AUC) for the model was 62%.

Conclusion: The severity of comorbidities and expected primary payer are the modifiable predictors identified to predict ESRD inpatient mortality. From this study, the findings suggest that strategies aimed at preventing the severity of comorbidities and ensuring universal health coverage might help reduce ESRD inpatient mortality in rural America.

## Introduction

Chronic kidney disease (CKD) is defined as the presence of kidney damage manifested by hematuria, albuminuria (albumin > 30 mg in 24 hours), or structural abnormalities, with an estimated glomerular filtration rate < 60 mL/minute/1.73 m^2^ persisting for more than three months [1]. End-stage renal disease (ESRD) is the terminal stage of CKD with an estimated glomerular filtration rate < 15 mL/minute/1.73 m^2^ and often requires renal replacement therapy such as dialysis and/or kidney transplant [1]. In the United States, the prevalence of CKD is estimated at 37 million [2]. Of this number, the prevalence of ESRD is estimated at more than 700,000 among American adults with the majority being on renal replacement therapy with 71% on dialysis and 29% with a kidney transplant [2]. The burden of CKD/ESRD gulps 33.8% of Medicare’s total fee-for-service spending and contributes to premature mortality in the United States.

Unfavorable clinical outcomes such as dialysis dependence, cardiovascular events, and all-cause mortality have been associated with the progression of CKD to ESRD [3,4]. A meta-analysis that included over 1.3 million participants showed that with decreasing kidney function, mortality among ESRD patients increased exponentially [5]. Some of the factors predicting mortality include age [6], absence of arteriovenous fistula [7], lack of early nephrology care [8], and comorbidities [9].

This study set out to identify the effect of comorbidities on mortality among ESRD inpatients in rural America. Our initial hypothesis was that the severity of comorbidities among ESRD inpatients is a predictor of mortality while adjusting for other potential confounders. The goal is that our study alongside others done on this subject will provide the background for building a model that may help predict mortality among ESRD inpatients. We also hope that the findings from this study could help institutions to address modifiable predictors of mortality identified in this study.

## Materials and methods

This is a cross-sectional study that used the 2016-2018 Nationwide Inpatient Sample (NIS) from the Healthcare Cost and Utilization Project (HCUP). The study included patients aged 18 years and older with ESRD hospitalized in rural hospitals in America. Independent variables from the survey included age (categorized into young, middle age, and elderly), gender, race, type of admission (elective versus nonelective), expected primary payer, type of hospital control, and severity of comorbidity (extrapolated from the Charlson Comorbidity Index with a score of 1-2 being mild, 3-4 being moderate, and greater than 4 being severe). The dependent variable was death while on admission. Univariate analysis was done using weighted and unweighted frequencies. Weighted bivariate analysis was done using Chi-square to see the significance of the association between independent and dependent variables. Only variables that were significant (P<0.05) on bivariate analysis were included in the logistic regression analysis. Regression analysis was also weighted. Both univariate and multivariate logistic regression analyses were done with the univariate logistic regression yielding the crude odds ratio and multivariate logistic regression yielding the adjusted odds ratio (AOR), with stepwise selection with entry and stay P-values of ≤0.05. The predictive validity of the model was assessed using the C-statistic. All analyses were done using the SAS Studio (SAS Institute Inc., Cary, NC, USA).

## Results

There were 144,575 hospitalizations in the study. Most (63.2%) ESRD admissions in the study were aged 60 years or more. More than half (58.5%) of all ESRD admissions were males. More than half (57.3%) were whites, and close to a third were blacks (32.3%). The majority (85.9%) of the admissions were nonelective. Medicare was the expected primary payer for 81% of the admissions. More than two-thirds (66.8%) of the hospitalizations were in private, not-for-profit hospitals. Just over half (52.4%) of the admissions had severe comorbidities (Table 1).

**Table 1:** Characteristics of the participants

Characteristics	Unweighted frequency (N=28,915)	Weighted frequency (N=144,575)	Weighted percentage
Age			
18-39	2,153	10,765	7.4
40-59	8,497	42,485	29.4
≥60	18,265	91,325	63.2
Sex			
Female	14,018	70,090	41.5
Male	14,897	74,485	58.5
Race			
White	16,579	82,895	57.3
Black	9,330	46,650	32.3
Hispanic	1,416	7,080	4.9
Asian or Pacific Islander	297	1,485	1.0
Native American	1,036	5,180	3.6
Other	257	1,285	0.9
Type of admission			
Elective	4,082	20,410	14.1
Nonelective	24,833	124,165	85.9
Expected primary payer			
Medicare	23,415	117,075	81.0
Medicaid	2,255	11,275	7.8
Private insurance	2,290	11,450	7.9
Self-pay	335	1,675	1.2
No charge	6	30	0.0
Other	614	3,070	2.1
Type of hospital control			
Government, nonfederal	4,538	22,690	15.7
Private, not-for-profit	19,318	96,590	66.8
Private, invest-owned	5,059	25,295	17.5
Comorbidities			
Mild	2,589	12,945	9.0
Moderate	11,183	55,915	38.7
Severe	15,143	75,715	52.4
Death during hospitalization			
Yes	1,437	7,185	5.0
No	27,478	137,390	95.0

On bivariate analysis analyzing the characteristics of respondents by death during hospitalization, there was a difference between the groups for the following characteristics: age group (P<0.0001), race (P<0.0001), type of hospital admissions (P<0.0001), expected primary payer (P<0.0001), type of hospital control (P<0.0001), and severity of comorbidities (P<0.0001). There was no difference between groups for gender (P=0.6577) (Table 2).

**Table 2:** Characteristics of the respondents by death during hospitalization

	Death during hospitalization		
Characteristics	Yes	No	P-value
Age			
18-39	215 (3.0)	10,550 (7.7)	<0.0001
40-59	1,255 (17.5)	41,230 (30.0)	
≥60	5,715 (79.5)	85,610 (62.3)	
Sex			
Male	3,720 (51.8)	70,765 (51.5)	0.6577
Female	3,465 (48.2)	6,6625 (48.5)	
Race			
White	4,705 (65.5)	78,190 (56.9)	<0.0001
Black	1,965 (27.4)	44,685 (32.5)	
Hispanic	210 (2.9)	6,870 (5.0)	
Asian or Pacific Islander	65 (0.9)	1,420 (1.0)	
Native American	190 (2.6)	4,990 (3.6)	
Other	50 (0.7)	1,235 (0.9)	
Type of admission			
Elective	1,185 (16.5)	19,225 (14.0)	<0.0001
Nonelective	6,000 (83.5)	118,165 (86.0)	
Expected primary payer			
Medicare	5,715 (79.5)	111,360 (81.1)	<0.0001
Medicaid	405 (5.6)	10,870 (7.9)	
Private insurance	590 (8.2)	10,860 (7.9)	
Self-pay	100 (1.4)	1,575 (1.2)	
No charge	10 (0.1)	20 (0.0)	
Other	365 (5.1)	2,705 (2.0)	
Type of hospital control			
Government, nonfederal	1,260 (17.5)	21,430 (15.6)	<0.0001
Private, not-for-profit	4,795 (66.7)	91,795 (66.8)	
Private, investor-owned	1,130 (15.7)	24,165 (17.6)	
Comorbidities			
Mild	455 (6.3)	12,490 (9.1)	<0.0001
Moderate	2,530 (35.2)	53,385 (38.9)	
Severe	4,200 (58.5)	71,515 (52.1)	

On multivariable logistic regression analysis, middle-aged and elderly patients had 40% (AOR: 1.40, 95% CI: 1.20-1.62) and 201% (AOR: 3.01, 95% CI: 2.61-3.48) more odds of mortality while hospitalized, respectively, compared to the young. Compared to whites, blacks had 19% (AOR: 0.81, 95% CI: 0.77-0.86) reduced odds of mortality, Hispanics had 47% (AOR: 0.53, 95% CI: 0.46-0.61) reduced odds of mortality, Native Americans had 27% (AOR: 0.73, 95% CI: 0.63-0.84) reduced odds of mortality, and Asian or Pacific Islanders had 30% (AOR: 0.70, 95% CI: 0.54-0.90) reduced odds of mortality. ESRD patients on nonelective hospitalization had 16% (AOR: 0.84, 95% CI: 0.79-0.90) reduced odds of mortality while hospitalized versus those on elective hospitalization. Compared to patients on Medicare, ESRD hospitalizations on Medicaid had 19% (AOR: 1.19, 95% CI: 1.06-1.32) higher odds of mortality, hospitalizations on private insurance had 26% (AOR: 1.26, 95% CI: 1.15-1.37) higher odds of mortality, self-pay patients had 99% (AOR: 1.99, 95% CI: 1.61-2.45) higher odds of mortality, and no charge patients had over 1400% (AOR: 15.61, 95% CI: 7.09-34.35) higher odds of mortality. Patients hospitalized in private, not-for-profit hospitals had 19% (AOR: 0.81, 95% CI: 0.76-0.86) reduced odds of mortality compared to those hospitalized in government hospitals, and those admitted in private, investor-owned hospitals had 24% (AOR: 0.76, 95% CI: 0.70-0.83) reduced odds of mortality compared to those admitted in government hospitals. ESRD patients with severe comorbidities had 40% (AOR: 1.40, 95% CI: 1.26-1.54) more odds of mortality compared to those with mild comorbidities, and those with moderate comorbidities had 22% (AOR: 1.22 95% CI: 1.10-1.36) more odds compared to those with mild comorbidities (Table 3).

**Table 3:** Predictors of inpatient mortality among ESRD inpatients in rural America Predictive validity of the model assessed using the C-statistic: 0.62

Characteristics	Crude OR (95%CI)	P-value	Adjusted OR	P-value
Age				
18-39	1 (ref)	<0.0001	1 (ref)	<0.0001
40-59	1.49 (1.09-2.05)		1.40 (1.20-1.62)	
≥60	3.27 (2.44-4.39)		3.01 (2.61-3.48)	
Race				
White	1 (ref)	<0.0001	1 (ref)	<0.0001
Black	0.73 (0.65-0.82)		0.81 (0.77-0.86)	
Hispanic	0.51 (0.38-0.69)		0.53 (0.46-0.61)	
Asian or Pacific Islander	0.76 (0.45-1.29)		0.70 (0.54-0.90)	
Native American	0.63 (0.46-0.87)		0.73 (0.63-0.84)	
Other	0.67 (0.34-1.32)		0.71 (0.53-0.94)	
Type of admission				
Elective	1 (ref)	0.0278	1 (ref)	<0.0001
Nonelective	0.82 (0.69-0.98)		0.84 (0.79-0.90)	
Expected primary payer				
Medicare	1 (ref)	<0.0001	1 (ref)	<0.0001
Medicaid	0.73 (0.58-0.92)		1.19 (1.06-1.32)	
Private insurance	1.06 (0.85-1.32)		1.26 (1.15-1.37)	
Self-pay	1.24 (0.78-1.97)		1.99 (1.61-2.45)	
No charge	9.74 (1.09-87.20)		15.6 1(7.09-34.35)	
Other	2.63 (1.95-3.55)		2.61 (2.33-2.93)	
Type of hospital control				
Government, nonfederal	1 (ref)	0.1037	1 (ref)	<0.0001
Private, not-for-profit	0.89 (0.75-1.05)		0.81 (0.76-0.86)	
Private, investor-owned	0.80 (0.64-0.98)		0.76 (0.70-0.83)	
Comorbidities				
Mild	1 (ref)	<0.0001	1 (ref)	<0.0001
Moderate	1.30 (1.04-1.63)		1.22 (1.10-1.36)	
Severe	1.61 (1.28-2.03)		1.40 (1.26-1.54)	

## Discussion

Among ESRD inpatients in rural America, the following variables have been identified as predictors of mortality: age, race, type of admission, expected primary payer, type of hospital control, and severity of comorbidities.

Expectedly, as patients age, the odds of mortality in ESRD inpatients increase. This could be explained by factors such as comorbidities that tend to accumulate as patients age, but even with adjustment for comorbidities, the effect of age as a predictor of mortality in ESRD inpatients remains. This effect of age on mortality in ESRD inpatients has been seen in another study [6,10].

Patients’ gender seems not to matter as a predictor of ESRD inpatient mortality as there was no difference between males and females on bivariate analysis; hence, gender was not included in the model of regression. The nonsignificance of gender has also been seen in other studies [11].

One of the highlights of our study is the paradoxical finding of lower odds of mortality among black and other minority patients with ESRD compared to their white counterparts. Given the better social determinants of health among whites compared to other minorities, it would be expected that the risk of mortality will be higher in minorities compared to whites. The opposite is however the case. This finding has been seen in other studies. Reasons to explain this paradox include survival bias, selection of healthier white patients for kidney transplantation, and a higher likelihood of renal replacement therapy in minorities with less severe comorbidities versus those with more severe comorbidities [12]. Other reasons that have been used to explain this paradox include the emigration of immigrant minorities to their countries of origin to die and differences in inflammation and nutrition [13], among others.

In terms of expected payer for the hospitalization, it appears that as patients receive less help in terms of organized health financing, the odds of mortality increase. It also appears that patients on government-financed insurance/assistance such as Medicare and Medicaid had reduced odds of mortality compared to self-pay/private insurance/no charge (largely charity) patients. In our study, after adjusting for other variables, Medicare and Medicaid patients had reduced odds of mortality compared to their counterparts who had private insurance, self-pay, or are supported by charities (no charge). This could be that the higher out-of-pocket cost (for private insurance or self-pay) or uncertainty of how to finance care (as in no charge patients) could cause less access to care, delay in seeking care, and other factors that could raise both morbidity and mortality in these patients. As a matter of fact, one study found that uninsured patients with end-stage renal disease begin dialysis later than insured patients and have poorer clinical outcomes by the time they start dialysis [14]. The implication of this is that measures aimed at achieving universal health coverage may greatly reduce inpatient mortality among ESRD patients.

Our study also showed that even after adjusting for age, as the severity of comorbidities among ESRD inpatients increases, the odds of mortality increase. The reason for this seems self-explanatory as severe comorbidities will have a more significant negative effect on the body physiology of patients compared to less severe comorbidities. This has been found in other studies [15,16]. The implication of this is that institutions should focus on developing strategies to reduce the accumulation of comorbidities as this may help in reducing mortality among ESRD inpatients.

Our study also showed that patients admitted into private hospitals (both investor-owned and not-for-profit) had reduced odds of mortality compared to those admitted into government, nonfederal hospitals. The reason for this is not exactly clear as there is a dearth of studies examining this subject in the literature. Possible reasons for this difference could be the resources available in terms of both humans and technology in caring for these patients, which may be better in private hospitals compared to government, nonfederal hospitals. This would be an interesting subject for future studies to examine.

Unexpectedly, patients with ESRD on nonelective admission had reduced odds of mortality compared to patients with elective admission. Again, there is a dearth of information in the literature on the effect of type of hospital admission (elective versus nonelective) on mortality in ESRD patients. This would constitute an interesting subject for future studies on this subject especially given that this finding goes against intuition.

Although the area under the curve (AUC) for our model is only 0.62, we hope that some of the variables used in our model can be used by other researchers in the future to create even more predictive models for mortality in ESRD inpatients.

Our study does have some limitations. First, although our study has found some association or relationship between the variables examined, this may not necessarily mean causation. Second, we only assessed the effect of variables that could be obtained in the NIS dataset; there are other variables such as the type of renal replacement therapy a patient is getting and the type of dialysis access that will predict mortality among ESRD inpatients, and this study did not adjust for all those other variables. Third, this study only included the rural population of the United States, and caution should be applied in applying the findings from this study to a different population.

## Conclusions

We hope that our research on this subject will provide the background for building a model that may help predict mortality among ESRD inpatients. We also hope that the findings from this study could help institutions address modifiable predictors of mortality identified in this study.

In conclusion, the NIS dataset provides a valuable tool for studying variables and mortality patterns among ESRD inpatients in the USA. We should bear in mind that not all variables are available for analysis from the NIS dataset, and this is a drawback of our study. We hope that other researchers will be able to use this concept in completing further research in this area, considering the racial differences in mortality comparing rural versus urban settings in the USA.
